# The New Incurable Wound

**DOI:** 10.3201/eid2209.AC2209

**Published:** 2016-09

**Authors:** Byron Breedlove, Paul M. Arguin

**Affiliations:** Centers for Disease Control and Prevention, Atlanta, Georgia, USA

**Keywords:** art science connection, emerging infectious diseases, art and medicine, about the cover, infectious diseases, antibiotics, antimicrobial resistance, Alexander the Great, Gordian Knot, Giovanni Paolo Panini, the new incurable wound, vedutisti, Panini projection, Alexander the Great Cutting the Gordian Knot, Chagas disease, public health

**Figure:**
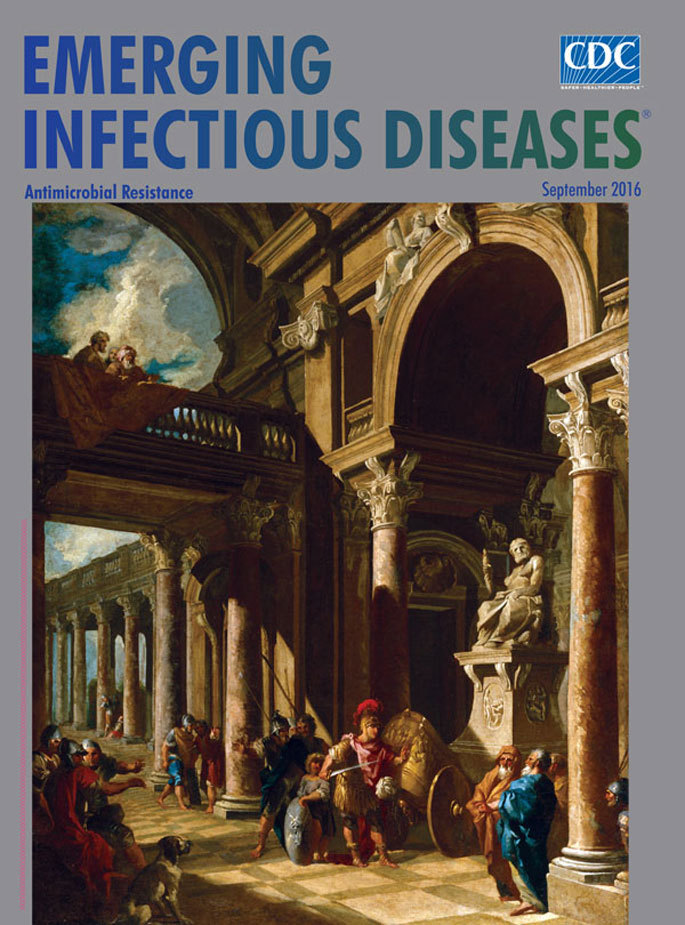
**Giovanni Paolo Panini (1691–1765), Alexander the Great Cutting the Gordian Knot (ca. 1718–1719). Oil on canvas, 28 7/8 in × 23 1/2 in/ 73.3 cm × 59.7 cm.** Public domain digital image courtesy of The Walters Art Museum, 600 N Charles St, Baltimore, Maryland, USA.

According to ancient mythology, the peasant Gordius, who married the fertility goddess Cybele, became king of Phrygia. He then dedicated his chariot to Zeus in the city Gordium and fastened it to a column with a large, complicated knot that became known as the Gordian knot. An oracle predicted that the future king of Asia would be the only person who could disentangle this knot.

Many individuals who traveled to Gordium attempted to untie the knot and thereby lay their claim to the throne, but their attempts proved futile. Then the Greek conqueror, Alexander the Great, whose actual name was Alexander III of Macedon, visited the city in 333 bce. He, too, was perplexed as he studied the knot, searching for its hidden ends. Whether prompted by impatience or insight, Alexander unexpectedly unsheathed his sword and sliced through the strands of rope, thereby severing and removing the knot. He subsequently conquered Asia, fulfilling the prophecy. He founded more than 70 cities and created a vast empire across three continents before his death in Babylon in June 323 bce.

Alexander’s bold, unexpected resolution gave rise to the oft-repeated saying, “cutting the Gordian knot.” That saying—now ubiquitously and inevitably linked to the shopworn notion of “thinking outside the box”—continues, however, to help codify thorny conundrums in multiple disciplines, including law, commerce, technology, education, economics, warfare, medicine, and health.

The depiction of Alexander’s eureka moment on this month’s cover was imagined by Giovanni Paolo Panini, “the most celebrated and popular view painter in eighteenth-century Rome,” according to the National Gallery of Art. Panini not only excelled as a *vedutisti*, he was also an architect and a professor of perspective and optics at the French Academy in Rome. He was considered a master of perspective, and his vistas of Rome, which featured many of the city’s antiquities, may have inspired creation of the Panini projection, a mathematical rule for constructing images with very wide fields of view, which was recently rediscovered and is now used in software for creating and viewing panoramic photographs.

Panini places Alexander in the center right of the bottom third of the painting, among a scattered group of onlookers. Some in the crowd, as well as a dog, watch with interest; others stand stiff and cross-armed—they have seen this act before. A child behind him holds his shield. The rows of columns and patterned floor add drama and perspective; the angled shadow cast by the balcony leads to Alexander, his sword glinting in the sunlight as he raises his left hand to warn away anyone who might step in for a closer look. A sculpture depicts Zeus perched on his stone throne, gripping his thunderbolt and peering directly at the viewer as if to say, “I knew this day would come.”

The interrelated, complex issues that have joined to create the current public health crisis of antimicrobial drug resistance constitute a Gordian knot as well. The question of whether we could see the rise of a postantibiotic period of infectious diseases that could mirror conditions of the preantibiotic and prevaccine period is not theoretical. Another Alexander, Sir Alexander Fleming, noted while accepting the 1945 Nobel Prize awarded for his 1928 discovery of penicillin that “It is not difficult to make microbes resistant to penicillin in the laboratory by exposing them to concentrations not sufficient to kill them, and the same thing has occasionally happened in the body.”

Howard Walter Florey, Ernst Boris Chain, and Norman Heatley subsequently recognized the potential of Fleming’s discovery and developed an effective drug from penicillin. Since the 1940s, antibiotics have greatly reduced illness and death from infectious diseases. But their widespread, and often inappropriate, use has come at a price: the infectious organisms have adapted to the antibiotics, making the drugs less effective.

Current events confirm Fleming’s prescience: bacterial infections incurable by antibiotics are now possible. Researchers found that a high proportion of swine-pathogenic *Escherichia coli* in Japan are resistant to colistin and noted concern for “a risk for transmission of *mcr-1* from these strains to human-pathogenic bacteria.” A recently published report describes a patient in the United States infected with *E. coli* containing the *mcr-1* resistance gene on a plasmid conferring resistance to colistin, the current antibiotic of last resort for treating patients with infections caused by some multidrug-resistant bacteria. Like Zeus, Fleming knew this day would come.

Some of the overlapping strands woven into the Gordian knot of antimicrobial resistance are myriad mutations and adaptations of various infectious organisms, lack of development of new antimicrobial agents, modern agricultural practice, and ineffective antibiotic stewardship. Tackling individual problems such as multidrug-resistant *Shigella* sp. infections, antibiotic overuse, or the transition of *Clostridium difficile* and *Staphylococcus aureus* from institutionally acquired to community-acquired infections is vital because an all-encompassing solution to the puzzle, such as that found by Alexander the Great, does not seem to be on our horizon.
